# Reported Symptoms Upon Exposure to Aeroallergen Highly Predict Allergen Sensitization Among 12‐Year‐Old Children

**DOI:** 10.1111/apa.70610

**Published:** 2026-05-28

**Authors:** E. Rönmark, L. Lundevall, A. Bjerg, R. Johansson, A. Winberg, L. Hedman

**Affiliations:** ^1^ Department of Public Health and Clinical Medicine, the OLIN and Sunderby Research Unit Umeå University Umeå Sweden; ^2^ Department of Clinical Sciences, Pediatrics Umeå University Umeå Sweden; ^3^ Barnläkarna I Täby Centrum BUUM Stockholm Sweden

**Keywords:** aeroallergen, allergy symptoms, children

## Abstract

**Aim:**

To evaluate the association between reported symptoms upon exposure to airborne allergens and sensitization to the corresponding allergen.

**Method:**

In a population‐based cohort, 1647 children aged 12 years were interviewed about symptoms from eyes, nose and lower airways upon exposure to birch, timothy, cat, dog, and horse, and were afterwards skin prick tested for the same allergens. Positive predictive value (PPV) was calculated for reported symptoms in relation to sensitization.

**Results:**

The prevalence of sensitization was generally higher in boys than girls, while reporting of symptoms was equal upon exposure. The proportion of sensitization among individuals reporting symptoms ranged from 70% to 95% and was highest for lower airway symptoms upon dog and cat exposure. Lower airway symptoms were the least prevalent but had the highest PPV for sensitization to each allergen (PPV = 75%–95%). The increasing number of symptoms when exposed to a specific allergen increased the likelihood of sensitization, and the PPV for three symptoms upon cat and dog exposure was > 95%.

**Conclusions:**

Self‐reported symptoms upon aeroallergen exposure highly predict sensitization to the same allergen. Unless allergen‐immunotherapy is indicated, objective measurements of sensitization may not always be necessary in 12‐year‐old children with a convincing history of airborne allergy.

## Summary

- Identification of specific IgE‐antibodies is essential in diagnosing allergies.
- In this large population‐based cohort study of 12‐year‐old children, reported allergy symptoms upon exposure to airborne allergens had very high positive predictive values (PPV) for IgE‐sensitization to the same allergen.
- 12‐year‐olds are old enough to be trusted in their reporting of symptoms, and unless allergen immunotherapy is indicated, objective measurements of sensitization may not always be necessary.

**Abbreviations**
HEP: histamine equivalent prick
IgE: immunoglobulin E
LAR: local allergic rhinitis
NPV: negative predicted value
OLIN: The Obstructive Lung Disease in Northern Sweden study
PPV: positive predicted value
SPT: skin prick test

## 1 Introduction

Allergic diseases constitute the largest group of non‐communicable diseases among children and teenagers. The prevalence has increased worldwide as well as in Sweden [1, 2, 3, 4, 5], and today almost 50% of young adults have or have had asthma or allergic rhinitis [6]. Even if some studies have found a levelling prevalence trend in asthma [1, 7, 8, 9] and allergic rhinitis [2, 8, 9, 10] during the last decades, allergic diseases represent a major clinical challenge, and the costs for both the affected individuals and for society are high [11].

Rhinitis and conjunctivitis in particular, as well as asthma, are all strongly associated with airborne allergen sensitization [6, 10, 12, 13]. Aeroallergen sensitization in early childhood is strongly related to development of allergic rhinitis and asthma in later childhood or young adulthood [14], and there is an association between early sensitization and asthma severity in children and teenagers [15]. Furthermore, remission from asthma is less common among sensitized individuals [6, 16].

Allergen sensitization can be diagnosed by direct measurement of specific IgE antibodies in sera or by indirect detection of specific IgE using skin prick test (SPT). Both tests are commonly used in clinical practice and in allergy research [17]. From a clinical point of view, identification of specific allergic sensitization is important, especially in asthma where allergic and non‐allergic phenotypes respond differently to treatments and have different prognoses [13, 16, 18]. Furthermore, allergen avoidance is only relevant in allergic individuals. Current guidelines point out the importance of identifying IgE sensitization and the correct allergen, and if immunotherapy is indicated, objective measure of specific IgE antibodies is mandatory [19, 20]. While numerous studies have investigated the prevalence of allergic symptoms among individuals sensitized to different allergens [6, 10, 12, 21, 22], the extent to which individuals reporting symptoms upon exposure to a specific allergen are sensitized to the same allergen is surprisingly poorly described.

The aim of this study was to evaluate the association between reported symptoms upon exposure to birch, timothy, cat, dog, and horse, and sensitization to the specific allergens in a cohort of 12‐year‐old children.

## 2 Materials and Method

### 2.1 Study Sample

Within the OLIN (Obstructive Lung Disease in Northern Sweden) studies several population‐based cohorts have been surveyed and followed longitudinally with questionnaires and allergy tests. The current study is based on the second OLIN paediatric cohort recruited in 2006 when all children in grade one and two (median age 8 years) in two municipalities in Norrbotten (Luleå and Kiruna), Sweden, were invited to a parental questionnaire and skin prick testing [23, 24]. The cohort was followed up and skin prick tested at age 12 years [25, 26]. Before skin prick testing, the children were interviewed about symptoms upon allergen exposure. The examinations were performed during January to April, before onset of the pollen season in the local area. Out of all invited, *n* = 1657 (85% of invited) participated in the SPT. All but 10 children also participated in the interview about symptoms, and these *n* = 1647 children constitute our study sample. The study was approved by the Regional Ethical Committee, Umeå, Sweden, Dnr 05‐157 M and 09‐206 M. Informed consent was collected from both parents/guardians and the participating child.

### 2.2 Skin Prick Test

The SPTs were performed at the children's schools and by a limited number of well‐trained research nurses. The SPT included the five allergens birch, timothy grass, cat, horse, and dog. In addition, mugwort, two dust mites, and two moulds were included in the SPT panel, but symptoms to these allergens were not evaluated. Allergen extracts from ALK, Hörsholm, Denmark, were used, and the procedure was performed according to current guidelines [27] by using a lancet with a 1 mm tip. The potency of the animal and pollen extracts was 10 HEP (histamine equivalent prick). In addition, a positive control (histamine 10 mg/mL) and a negative control were included. A mean wheal size ≥ 3 mm after 15 min was regarded as a positive test.

### 2.3 The Interview and the Definitions of Clinical Symptoms

The interview was performed before the SPT and by the same nurse that did the SPT, and without any assistance from a parent. The child was asked about allergic symptoms from the eyes, nose, and lower airways upon contact with five common airborne allergens: birch, timothy grass, cat, horse, and dog. Pollen allergens have distinctive seasons in northern Sweden, and if needed we clarified that birch pollen is relevant during spring/early summer and grass during later summertime. The full interview questions are presented in Table S1 and are defined as follows:

Eye symptoms: itchy or runny eyes upon exposure to each of the five allergens.

Nose symptoms: congested and/or runny nose when exposed to each of the five allergens.

Lower airway symptoms: wheeze or shortness of breath, that is, symptoms of asthma, when exposed to each of the five allergens.

### 2.4 Statistical Analysis

All statistical analysis was performed using IBM SPSS Statistics version 24. Difference between proportions was calculated by Pearson's Chi‐Square Test. A *p*‐value < 0.05 was defined as statistically significant. Symptoms from eyes, nose, and lower airways were analysed in relation to sensitization to allergens from birch, grass, cat, dog, and horse, respectively. Analysis of positive predictive value (PPV) was calculated according to the formula $a/\left(a+b\right)$ where “$a^{"}$ is children with symptoms who were sensitized, and “$b^{"}$ is children with symptoms who were not sensitized.

## 3 Results

### 3.1 Prevalence of Allergic Sensitization

The prevalence of a positive SPT for any of the 10 allergens was 41.4%, with a higher prevalence among boys than girls, 45.7% vs. 37.0% (*p* < 0.001). Sensitization to cat was most common, 26.4%, followed by sensitization to dog, 22.6%. Sensitization to timothy and birch was similarly prevalent, 22.2% and 21.3%. Sensitization to dust mites and moulds was low, < 3% (Table 1).

**TABLE 1:** Prevalence (%) of allergen sensitization by sex in the cohort.

Allergen	Sex		*p*	Total (*n* = 1657)
	Girl	Boy		
	(*n* = 814)	(*n* = 843)		
Birch	19.8	22.8	0.136	21.3
Timothy Grass	19.3	25.0	0.005	22.2
Mugwort	4.4	5.6	0.285	5.0
Cat	23.5	29.6	0.005	26.4
Horse	11.2	13.9	0.095	12.5
Dog	20.1	25.1	0.017	22.6
Any mite	1.4	2.1	0.224	1.8
Any mould	2.5	3.3	0.294	2.9
Any allergen	37.0	45.7	< 0.001	41.4

### 3.2 Prevalence of Clinical Symptoms

Among all children, the prevalence of any symptom when exposed to any of the five pollen or animal allergens was 33.4% (*n* = 686). For all allergen exposures, eye‐ and nose symptoms were the most common. Eye symptoms were the most prevalent symptom upon exposure to cat, 18.2%. No significant difference in the prevalence of exposure‐related symptoms was observed when comparing boys and girls (Table 2).

**TABLE 2:** Prevalence (%) of symptoms from eyes, nose and lower airways when exposed to different allergens among all children in the cohort and by sex.

Allergen	Symptom	Sex		*p*	Total (*n* = 1647)
		Girl	Boy		
		(*n* = 811)	(*n* = 836)		
Birch	Eyes	15.3	15.1	0.902	15.2
	Nose	15.2	14.0	0.500	14.6
	Lower airways	3.1	2.5	0.485	2.8
Timothy	Eyes	10.2	12.3	0.181	11.3
	Nose	11.0	10.9	0.947	10.9
	Lower airways	2.0	1.4	0.397	1.7
Cat	Eyes	18.4	18.1	0.861	18.2
	Nose	14.6	13.2	0.408	13.9
	Lower airways	5.6	5.0	0.630	5.3
Horse	Eyes	10.7	8.9	0.200	9.7
	Nose	8.8	7.5	0.366	8.1
	Lower airways	5.1	3.6	0.143	4.3
Dog	Eyes	9.0	11.0	0.173	10.0
	Nose	7.9	8.4	0.715	8.1
	Lower airways	3.7	4.2	0.608	3.9

### 3.3 Prevalence of Sensitization Among Individuals With Symptoms

**FIGURE 1:**
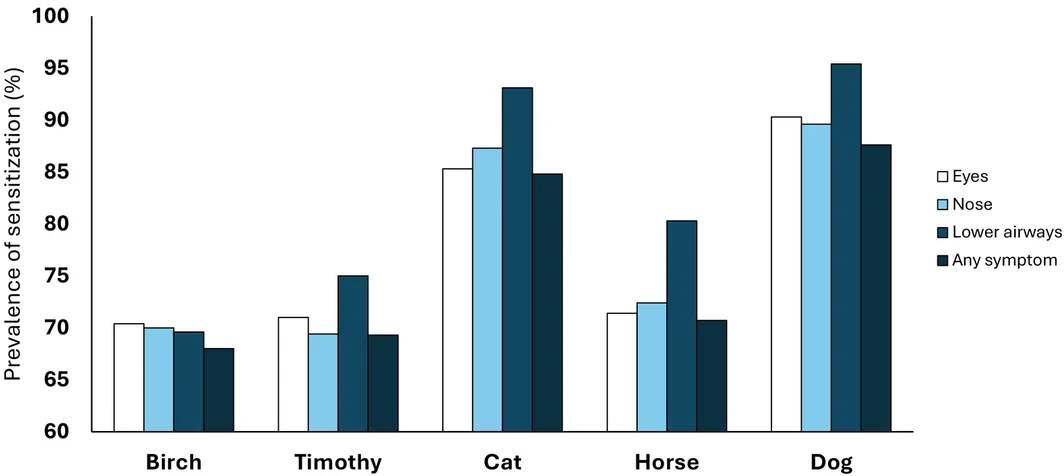
Prevalence (%) of sensitization to the specific allergen by type of reported symptoms upon exposure to the same allergen. The y‐axis starts at 60% and not 0%.

For all five allergens, 70.4%–95.4% of those who reported symptoms upon allergen exposure were also sensitized to the corresponding allergen. The proportion of sensitized individuals varied more between different allergens than between different symptoms. The strongest association was found for cat and dog exposure: 85.3%–93.1% of those reporting symptoms to cat, and 90.3%–95.5% of those reporting symptoms to dog were sensitized (Figure 1).

For all allergens except birch, lower airway symptoms upon exposure showed the highest prevalence of sensitization (75% to 95%), followed by eye symptoms upon dog exposure (90%) and nose symptoms upon cat exposure (87%). The association between symptoms and sensitization to pollens was weaker: the prevalence of sensitization to birch was 68.0% among individuals with any symptoms to birch, and 69.3% of those with any symptoms to timothy (Figure 1).

### 3.4 Prevalence of Symptoms Among Sensitized and Non‐Sensitized Individuals

Far from all children sensitized to a specific allergen reported symptoms upon allergen exposure: as many as 35.5%–59.8% of sensitized children had no symptoms (Table 3). The highest prevalence of any symptom was found among those sensitized to cat, 64.5%, and the lowest, 40.8%, among those sensitized to timothy. For all allergen exposures, eye symptoms were the most often reported, followed by nose symptoms. Among individuals sensitized to pollen, boys and girls reported symptoms to an equal extent, while among those sensitized to cat and horse significantly more girls than boys reported symptoms upon exposure to the respective allergens. The prevalence of reported symptoms among non‐sensitized individuals was low and similar in boys and girls.

**TABLE 3:** Prevalence (%) of symptoms when exposed to the specific allergen among sensitized and non‐sensitized to the same allergen, and by sex.

Allergen	Symptom	Sensitized to the specific allergen				Non‐sensitized to the specific allergen			
		Girls	Boys	*p*	All	Girls	Boys	*p*	All
Birch	Eyes	52.8	48.2	0.385	50.3	6.1	5.3	0.503	5.7
	Nose	49.1	47.2	0.753	48.1	6.9	4.2	0.032	5.6
	Lower airways	11.3	7.4	0.203	9.2	1.1	1.1	0.984	1.1
	Any symptom	56.6	54.2	0.654	55.3	8.1	5.9	0.113	7.0
Timothy	Eyes	33.5	38.1	0.372	36.2	4.7	3.7	0.349	4.2
	Nose	35.5	33.3	0.669	34.2	5.2	3.4	0.105	4.3
	Lower airways	6.5	5.2	0.623	5.8	0.9	0.2	0.125	0.5
	Any symptom	40.0	41.4	0.784	40.8	5.9	4.3	0.186	5.1
Cat	Eyes	65.8	53.8	0.012	59.0	4.2	3.1	0.301	3.6
	Nose	53.5	40.1	0.006	45.9	2.9	1.9	0.247	2.4
	Lower airways	22.5	15.8	0.077	18.7	0.5	0.5	1.000	0.5
	Any symptom	70.6	59.9	0.021	64.5	4.7	3.6	0.344	4.1
Horse	Eyes	65.2	49.1	0.022	56.1	4.0	2.4	0.074	3.2
	Nose	52.8	43.1	0.168	47.3	3.3	1.8	0.069	2.6
	Lower airways	34.8	22.4	0.049	27.8	1.4	0.6	0.109	1.0
	Any symptom	71.9	55.2	0.014	62.4	4.9	2.5	0.018	3.7
Dog	Eyes	40.6	40.2	0.933	40.4	1.2	1.3	0.935	1.3
	Nose	35.6	30.1	0.265	32.5	1.1	1.1	1.000	1.1
	Lower airways	18.1	15.8	0.552	16.8	0.2	0.3	0.613	0.2
	Any symptom	47.5	44.5	0.566	45.8	1.8	1.9	0.920	1.9

*Note:* *p*‐value represent comparison between girls and boys.

### 3.5 Significance of Number of Symptoms

**FIGURE 2:**
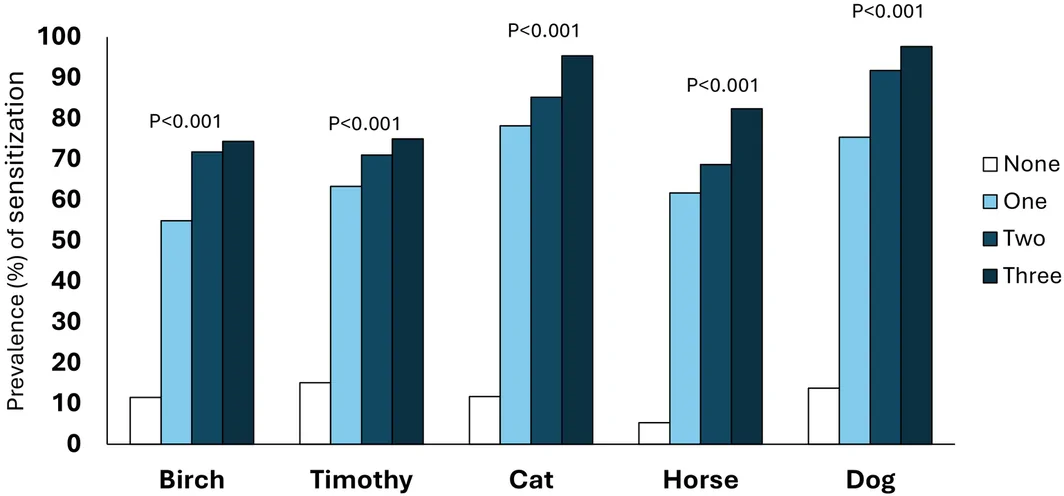
Prevalence (%) of sensitization to the specific allergen by number of reported symptoms upon exposure to the same allergen. Difference between groups analysed by Chi square test.

Reporting two symptoms when exposed to any of the different allergens was most common (ranging from 5.0% for horse to 10.6% for birch) compared to reporting only one symptom (from 2.9% for horse to 6.7% for cat), or three symptoms (from 1.5% for timothy grass to 3.9% for cat). This pattern was similar for all allergens and similar in boys and girls. The number of reported symptoms was associated with sensitization, with a sensitization prevalence of 5%–15% in individuals with no symptoms compared to 74%–98% in individuals with all three symptoms (Figure 2). The most convincing trends were seen for symptoms to dog and cat allergens, with 97.7% of individuals reporting three symptoms to dog and 95.4% reporting three symptoms to cat being sensitized. Only 2.3% (*n* = 1) and 4.6% (*n* = 3) respectively reported three symptoms to cat and dog without being sensitized. The PPV of reporting any symptom was 84.8% for cat and 87.6% for dog exposure, while if all three symptoms were present, the corresponding values were 95.4% and 97.7%, respectively. The specificity of reporting three symptoms was very high: 99.2%–99.9% (Table 4).

**TABLE 4:** Positive (PPV) and negative (NPV) predictive value, sensitivity and specificity (%) of reporting any symptom and all three symptoms for the specific allergen, in relation to sensitization to the specific allergen.

	Any symptom^a^					All three symptoms^a^				
	Birch	Timothy	Cat	Horse	Dog	Birch	Timothy	Cat	Horse	Dog
PPV	68.0	69.3	84.8	70.7	87.6	74.4	75.0	95.4	82.4	97.7
NPV	11.5	15.1	11.7	5.3	13.8	11.5	15.1	11.7	5.3	13.8
Sensitivity	55.3	40.8	64.5	62.4	45.8	15.6	7.7	28.7	35.3	17.4
Specificity	93.0	94.9	95.9	96.3	98.1	99.2	99.5	99.7	99.4	99.9

^a^ Symptoms from eye, nose, or lower airway.

## 4 Discussion

The association between symptoms upon allergen exposure and sensitization to the specific allergen was generally high but varied between allergens. Overall, more than two‐thirds of individuals reporting symptoms upon exposure to a specific allergen showed a corresponding sensitization, and the strongest association was found for cat and dog allergens. The presence of a symptom triad including eye, nose, and lower airway symptoms further strengthened the association. These findings support the widely used clinical practice to diagnose allergies without the use of confirmative IgE tests when a convincing detailed history of symptoms upon allergen exposure is present. We can also conclude that 12‐year‐olds are old enough to be trusted in their reporting of symptoms without the assistance of a parent.

In line with other studies [10, 28, 29, 30], we found a higher prevalence of sensitization among boys than girls. Thus, one would expect a higher prevalence of reported symptoms upon specific allergen exposure in boys, but no significant sex differences were found. However, there was a trend that girls more often reported symptoms to horse, despite a tendency to higher sensitization to horse in boys. Furthermore, in the analyses stratified by sensitization status, significantly more girls sensitized to cat and horse reported symptoms when exposed to the corresponding allergen. This finding could partly be caused by a reporting bias, since more girls than boys participate in horse‐related sports and activities in Sweden and thus have more contact with horses. In our cohort, 25% of girls compared with 2% among boys reported horse riding at age 8 years (data not published). Why girls sensitized to cat report more clinical symptoms upon cat exposure compared to boys is difficult to explain, but it may reflect how children at this age interact with pets. These sex differences may also be associated with type 1 error due to multiple testing. However, previous publications from the current cohort and other cohorts have confirmed higher incidence and prevalence of asthma in girls compared with boys during late teens [30, 31, 32]. Taken together, it seems that girls are more prone to report clinical symptoms and the reason for this needs further investigation.

To our knowledge, there are no publications based on an unselected population sample investigating the proportion of individuals reporting symptoms to specific allergen exposure in relation to sensitization to the same allergen. Instead, several studies have investigated the association between asthma and rhinitis and different types of sensitization. These studies have shown that allergen sensitization in general, and sensitization to pollen in particular, is more strongly associated with rhinitis than with asthma [6, 10, 12, 21, 22]. This is thought to reflect the larger size of pollen particles compared with pet allergens, which makes them impact mainly in the upper airways, including the eyes. Our study was conducted from the perspective of a typical clinical consultation as we evaluated common clinical symptoms in relation to SPT and found that symptoms upon exposure to the most common aeroallergens in our region had a very high specificity as well as a high PPV. However, the PPV for symptoms upon exposure differed between allergens: the higher PPV for symptoms upon cat and dog exposure compared with pollens may be explained by the fact that exposure from a specific animal is easier to identify compared with exposure to a specific pollen during the pollen season when a lot of potential allergens are in circulation at the same time as birch and timothy grass respectively. The lack of dust mites in our area and the low numbers of children sensitized to dust mite [10] is a favouring factor as the reports of symptoms from indoor allergens, that is, cat and dog, are not biassed by occurrence of mite allergens indoors. The lower PPV for pollen induced symptoms may also be associated with recall bias as the examination and interview were performed outside of the pollen season.

In the current study, we used SPT to identify aeroallergen sensitization and we defined a positive test as a wheal size of 3 mm or more. In general, results based on SPT and specific serum IgE correlate well [17, 21, 23, 33], with IgE in sera being more easily quantified and SPT considered more sensitive [17]. Furthermore, there is no gold standard regarding the cut‐off for clinical significance for neither serum IgE nor wheal size in SPT [34]. However, higher levels of specific IgE are associated with more severe disease and multimorbidity [35, 36, 37]. A study evaluating SPT and serum IgE against clinical symptoms reported that SPT wheal size with a cut‐off at 3 mm had a higher specificity for asthma and hay fever than IgE with a cut‐off of 0.35 IU/mL, while the sensitivity did not differ [33].

Even if the proportion of non‐sensitized individuals who reported symptoms upon allergen exposure was low, it is worth commenting on. The use of SPT may have resulted in some false positive or negative results despite the good correlation with s‐IgE as discussed above. Another possible source of error is that symptoms in some cases could have been attributed to the wrong allergen. This could be the case when symptoms appeared in environments where several different allergens were present, such as during the pollen season as described above, or in homes or stables that host several different animals and, in the case of stables, additionally contain allergens from hay and irritants. There are also the non‐IgE mediated equivalents to allergic rhinitis, conjunctivitis, and asthma where less known immunological mechanisms may play a role in the mediation of hypersensitivity [38], as well as local allergic rhinitis (LAR) [39]. The low sensitivity of the interview questions was expected and is explained by the fact that many children that were sensitized at age 12 years had not yet developed clinical symptoms, even if most children with sensitization in early school age sooner or later will develop either allergic rhinitis or asthma [12, 14].

The large population‐based study design with a high participation rate minimized selection bias, which contributes to the validity of the results. Moreover, the SPT has been validated against serum‐IgE levels in a random sample (*n* = 696, 76% of invited), and the agreement with the results based on SPT was excellent [25]. Finally, and importantly, the interviews took place before the SPTs so that symptom report bias related to the SPT result was avoided. Our results were in accordance with the findings from other studies regarding the prevalence of both asthma and allergic sensitization [12, 39, 40, 41] which strengthens the external validity of our results.

Limitations include recall bias and limited exposure to some of the specific allergens, particularly horses, as discussed above. However, the agreement between the parents' and the children's answers has been evaluated and there was a strong agreement between self‐completed and parentally completed questionnaires, particularly regarding questions about asthma and rhinitis, but also regarding exposures [42, 43]. Thus, accurate reports of symptoms need not be mistrusted based on the subjects' age or limited experience. Symptoms are more likely to appear the longer a person has been sensitized [14], and it would be interesting to explore the concurrence of sensitization and symptoms in older subjects. We have not in detail explored which symptoms are more likely to co‐exist, but it is known that rhinitis without conjunctivitis has little relevance in the context of allergy, and these two were also, separately, the most common symptoms of the three.

## 5 Conclusion

In this population‐based study, we found that most children reporting symptoms upon exposure to a specific airborne allergen were also sensitized to that allergen. We found a significant association between the number of symptoms and allergic sensitization. Especially for cat and dog allergens, the PPV of reporting three symptoms was high (> 95%). This may prove useful in the clinic, as cats and dogs are our most common pets, and children reporting two or three symptoms when exposed to these allergens will not necessarily need to be tested for sensitization unless allergen immunotherapy is indicated.

## Author Contributions

**L. Hedman:** investigation, conceptualization, writing – review and editing, methodology, validation, visualization, project administration, supervision. **R. Johansson:** conceptualization, investigation, writing – review and editing, methodology, validation, visualization. **A. Winberg:** conceptualization, investigation, writing – review and editing, methodology, validation, visualization. **L. Lundevall:** conceptualization, writing – original draft, methodology, validation, visualization, writing – review and editing, formal analysis. **E. Rönmark:** conceptualization, investigation, funding acquisition, methodology, validation, visualization, formal analysis, project administration, data curation, supervision, resources, writing – original draft, writing – review and editing. **A. Bjerg:** conceptualization, investigation, writing – review and editing, methodology, validation, visualization.

## Funding

Financial support was received from The Swedish Heart Lung Foundation, The Swedish Asthma‐Allergy Foundation, The Swedish research council, a regional agreement between Umeå University and Västerbotten County Council (ALF), VISARE NORR fund: Northern County Councils Regional Federation, and Norrbotten County Council. Additional support was provided from ALK, Denmark. The funders had no role in the design, data collection, data analysis, or reporting of this study.

## Conflicts of Interest

The authors declare no conflicts of interest.

## Supporting information


**Table S1:** The interview questions regarding clinical symptoms upon allergen exposure. Eye symptoms: Do you get symptoms from the eyes, itchy or runny eyes, when you are in contact with dog/cat/horse/birch/grass? Nose symptoms: Do you get symptoms from the nose, congested and/or running nose, when you are in contact with dog/cat/horse/birch/grass? Lower airway symptoms: Do you get symptoms from the airways, wheeze or shortness of breath, that is, symptoms of asthma, when you are in contact with dog/cat/horse/birch/grass? For pollen, and if necessary, we clarified that birch pollen is relevant during spring/early summer and grass during later summertime.

## Data Availability

The data that support the findings of this study are available on request from the corresponding author. The data are not publicly available due to privacy or ethical restrictions.
