# Supplementary material for: Reported Symptoms Upon Exposure to Aeroallergen Highly Predict Allergen Sensitization Among 12‐Year‐Old Children
Source: Acta Paediatr. 2026 May 28;115(10):2125–32. doi: 10.1111/apa.70610 (PMC13552595; doi:10.1111/apa.70610)
Supplement: Supplementary file 1 [file APA-115-2125-s001.docx]

**Table S1. The interview questions regarding clinical symptoms upon allergen exposure.**

**Eye symptoms:** Do you get symptoms from the eyes, itchy or runny eyes, when you are in contact with dog/cat/horse/birch/grass?

**Nose symptoms**: Do you get symptoms from the nose, congested and/or running nose, when you are in contact with dog/cat/horse/birch/grass?

**Lower airway symptoms:** Do you get symptoms from the airways, wheeze or shortness of breath, i.e. symptoms of asthma, when you are in contact with dog/cat/horse/birch/grass?

For pollen, and if necessary, we clarified that birch pollen is relevant during spring/early summer and grass during later summertime.
